# Expression Analysis of Zinc Transporters in Nervous Tissue Cells Reveals Neuronal and Synaptic Localization of ZIP4

**DOI:** 10.3390/ijms22094511

**Published:** 2021-04-26

**Authors:** Chiara A. De Benedictis, Claudia Haffke, Simone Hagmeyer, Ann Katrin Sauer, Andreas M. Grabrucker

**Affiliations:** 1 Cellular Neurobiology and Neuro-Nanotechnology Lab, Department of Biological Sciences, University of Limerick, V94PH61 Limerick, Ireland; chiara.debenedictis@ul.ie (C.A.D.B.); claudia.haffke@gmx.de (C.H.); simone.hagmeyer@gmail.com (S.H.); ann.katrin.sauer@ul.ie (A.K.S.); 2 Bernal Institute, University of Limerick, V94PH61 Limerick, Ireland; 3 Health Research Institute (HRI), University of Limerick, V94PH61 Limerick, Ireland

**Keywords:** Zinc, ZnT, ZIP, glia, SLC30, SLC39A4, brain, synapse

## Abstract

In the last years, research has shown that zinc ions play an essential role in the physiology of brain function. Zinc acts as a potent neuromodulatory agent and signaling ions, regulating healthy brain development and the function of both neurons and glial cells. Therefore, the concentration of zinc within the brain and its cells is tightly controlled. Zinc transporters are key regulators of (extra-) cellular zinc levels, and deregulation of zinc homeostasis and zinc transporters has been associated with neurodegenerative and neuropsychiatric disorders. However, to date, the presence of specific family members and their subcellular localization within brain cells have not been investigated in detail. Here, we analyzed the expression of all zinc transporters (ZnTs) and Irt-like proteins (ZIPs) in the rat brain. We further used primary rat neurons and rat astrocyte cell lines to differentiate between the expression found in neurons or astrocytes or both. We identified ZIP4 expressed in astrocytes but significantly more so in neurons, a finding that has not been reported previously. In neurons, ZIP4 is localized to synapses and found in a complex with major postsynaptic scaffold proteins of excitatory synapses. Synaptic ZIP4 reacts to short-term fluctuations in local zinc levels. We conclude that ZIP4 may have a so-far undescribed functional role at excitatory postsynapses.

## 1. Introduction

Zinc is one of the most prevalent trace metals in the human brain, where it is found in its free (aqueous) ionic form within cells and inside neurotransmitter vesicles and its protein-bound form. Through its function, acting as a modulator of neurotransmission, signaling ion, and structural or catalytic part of proteins, zinc is vital for several processes, such as neurogenesis, neuronal migration, and differentiation, as well as neurotransmission and synaptic plasticity [1]. Two different zinc-transporter families regulate cellular zinc homeostasis: the Irt-like protein (ZIP) family (SLC39A) and zinc transporter (ZnT) family (SLC30A). While SLC39As mediate the influx of zinc into the cytosol from the extracellular space and the lumen of intracellular compartments, SLC30A family members facilitate the removal of zinc from the cytosol, either out of the cell or into vesicles and organelles.

The particular function of several of the ten ZnT and fourteen ZIP family members have been described in the brain so far. For example, ZnT3 has been identified as a major transporter for loading synaptic vesicles with zinc [2], and ZnT1 was shown tightly associated with NMDA receptors at postsynaptic densities [3]. The expression of ZIP12 is reported in the brain of different species, such as humans, mice, and frogs, where ZIP12 has a role in neurodevelopment [4].

However, little is known about the function of other zinc transporters in the brain, such as ZIP4, although the expression of *Zip4* has been reported on mRNA level in the choroid plexus, brain capillaries [5], and gliomas [6,7]. ZIP4 was initially identified as zinc-uptake transporter in the small intestine. There, ZIP4 is dynamically regulated by different mechanisms depending on zinc availability [8]. In mice, low zinc levels lead to increased *Zip4* mRNA expression and ZIP4 localization to the apical surface of enterocytes [9]. In contrast, in the presence of zinc, membrane-bound ZIP4 levels are reduced through two processes that are dependent on zinc levels [10]. At physiological zinc concentrations, ubiquitination-independent constitutive endocytosis occurs, while at high zinc concentration, ubiquitination-dependent degradation takes place [10]. Post-translational regulation of ZIP4 may contribute to endocytosis and recycling [11]. The expression levels, in turn, may be regulated by improved mRNA stability [12] and activity of the zinc-finger transcription factor Krüppel-like factor 4 (KLF4) [13]. While the transcriptional regulation is slow, endocytosis and degradation are rapid adjustments to changes in zinc levels that may take place within minutes.

Loss of function mutations in *Zip4* cause acrodermatitis enteropathica (AE) [14], a rare inherited defect in dietary zinc absorption. This disorder, if untreated, results in systemic zinc deficiency, and patients have been reported to suffer from skin lesions, alopecia, diarrhea, and other gastrointestinal symptoms, but also neurological symptoms, such as fatigue, moodiness, irritability, photophobia, and anorexia. In the advanced stage, growth delay, immune deficiency, photophobia, mental slowing, psychical disturbances of the schizoid type, and depression may occur [15,16,17,18,19,20,21,22,23,24]. These symptoms might be caused by a systemic zinc deficiency, but also might also be caused by a specific function of *Zip4* in the brain.

It has been shown that the expression of zinc transporters is altered in several brain diseases. For example, modified protein and mRNA levels of ZIP1, ZnT1, ZnT4, ZnT6, and ZnT10 have been reported in Alzheimer’s disease (AD) [25,26], and elevated levels of ZnT6 in Pick’s disease [27]. Moreover, increased cortical expression of ZIP12 has been found in schizophrenia [28]. However, a complete expression profile and subcellular distribution of zinc transporters in neural cells have not been described in detail so far.

Here, we report the expression profile of zinc transporters in the hippocampus of rats in vivo and rat hippocampal neurons and astrocytes in vitro. Given that little is known about the expression and localization of ZIP4 in the brain and its function and that ZIP4, we investigated this zinc transporter in more detail. We identified ZIP4 as novel neuronal zinc transporter that localizes to the cell soma but also synapses. At synapses, ZIP4 is found in a complex with SH3 and multiple ankyrin repeat domains 3 (SHANK3), an essential scaffold protein in the postsynaptic density of excitatory glutamatergic synapses and reacts to transient changes in zinc levels.

## 2. Results

### 2.1. Expression of Zinc Transporters in Adult Rat Brain and Rat Neurons and Astrocytes on mRNA Level

**Figure 1:**
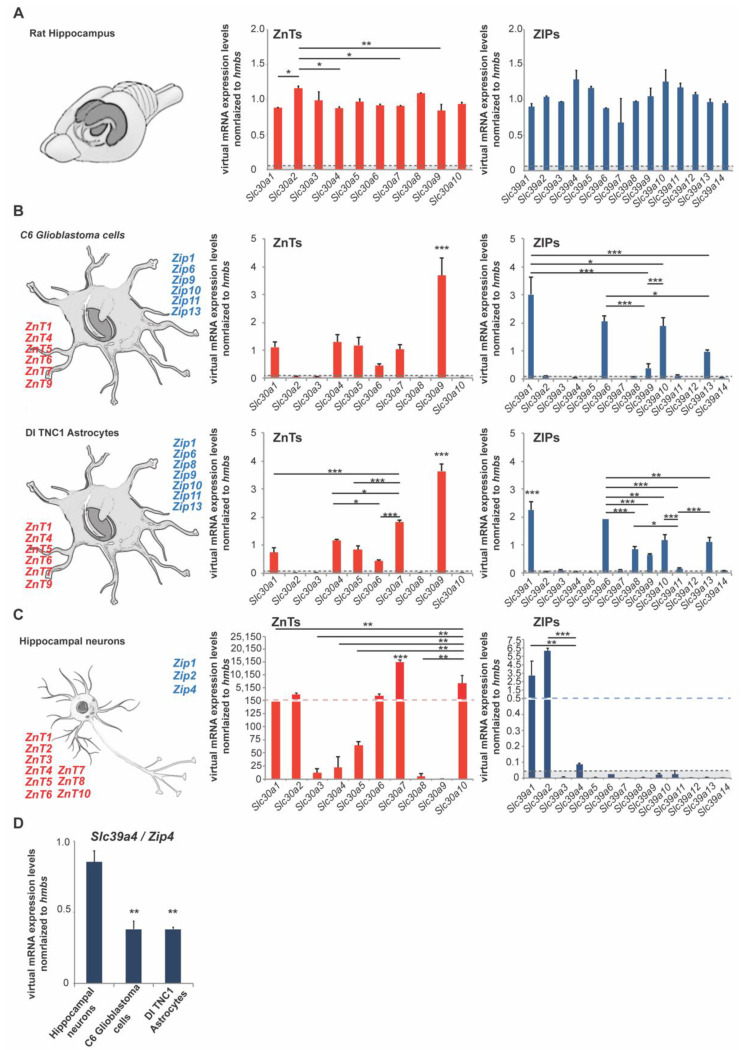
mRNA expression of zinc transporters in adult rat brain and rat neurons and astrocytes. mRNA expression was analyzed by qRT-PCR. mRNA levels are shown as average virtual mRNA concentration normalized to *hmbs* from three biological replicates measured in technical triplicates. (**A**) Hippocampal brain mRNA lysate was prepared from rats. The expression of all zinc transporters was detected in brain lysate. For a detailed statistical analysis, please see Supplementary Table S1A. (**B**) mRNA lysate was prepared from C6 glioblastoma cells and DI TNC1 astrocytes. Significant differential expression of zinc transporters was detected in both cell lines (one-way ANOVA, *p*_Glioblastoma_ ≤ 0.0001, *p*_DI TNC1_ ≤ 0.0001). The highest expression was detected for *ZnT1, ZnT4, ZnT5, ZnT6, ZnT7, ZnT9*, and *Zip1, Zip6, Zip9, Zip10, Zip11, Zip13*. *Zip8* expression was significantly higher in C6 glioblastoma cells (*t*-test: *p* = 0.038). For a detailed statistical analysis, please see Supplementary Table S1B. (**C**) mRNA lysate was prepared from primary hippocampal neurons. Significant differential expression of zinc transporters was detected (one-way ANOVA, *p* ≤ 0.0001). The highest expression was detected for *ZnT1, ZnT2, ZnT6, ZnT7, ZnT10*, and *Zip1, Zip2 Zip4*. For a detailed statistical analysis, please see Supplementary Table S1C. (**D**) *Zip4* expression was significantly higher in neurons compared to both astrocyte cell lines (one-way ANOVA, *p* = 0.002; Tukey’s post hoc test: neurons vs. C6: *p* = 0.009; neurons vs. DI TNC1: *p* = 0.0048). Only expression 5% above of *hmbs* expression was considered for the analysis (dotted line **A**–**C**).

In the first set of experiments, we performed an expression analysis of ZnTs (*Slc30a1–Slc30a10*) and ZIPs (*Slc39a1–Slc39a14*) using rat hippocampus lysate from three animals (three female adult rats). The results show the expression of all zinc transporters can be detected to some extent in the hippocampus. For the zinc transporters of the ZnT family, we noticed the highest expression on mRNA levels for *Slc30a2* (*ZnT2*), *Slc30a3* (*ZnT3*), *Slc30a5* (*ZnT5*), *Slc30a8* (*ZnT8*), and *Slc30a10* (*ZnT10*) with significant differences among means (one-way ANOVA: *p* = 0.0049); post hoc tests were reported in the supplementary file. Regarding the zinc transporters of the ZIP family, we found the highest expression for the following transporters: *Slc39a4* (*Zip4*), *Slc39a5* (*Zip5*), *Slc39a10* (*Zip10*), *Slc39a11* (*Zip11*), and *Slc39a12* (*Zip12*) without any significant differences among means (one-way ANOVA: *p* = 0.0576) (Figure 1A).

To further investigate to what extent neurons and glial cells (astrocytes) are contributing to the overall expression, we used primary hippocampal neurons from rats as well as two rat astrocyte cell lines (C6 glioblastoma cells and DI TNC1 astrocytes) (Figure 1B,C). The expression profiles were very consistent between both astrocyte cell lines. In both cell lines, we detected significant expression of *Slc30a1* (*ZnT1*), *Slc30a4* (*ZnT4*), *Slc30a5* (*ZnT5*), *Slc30a6* (*ZnT6*), *Slc30a7* (*ZnT7*), *Slc30a9* (*ZnT9*), and *Slc39a1* (*Zip1*), *Slc39a6* (*Zip6*), *Slc39a9* (*Zip9*), *Slc39a10* (*Zip10*), *Slc39a11* (*Zip11*), *Slc39a13* (*Zip13*) (one-way ANOVA: *p* < 0.0001) (Figure 1B). The expression of *Slc39a8* (*Zip8*) was detected in DI TNC1 astrocytes, but significantly (unpaired *t*-test: *p* = 0.0038) less expression was detected in C6 glioblastoma cells, a finding that may have relevance for the pathomechanisms of glioblastomas (Figure 1B). The zinc transporter ZIP8 has already been associated with neuroblastomas and urothelium and urothelial cancers in humans [29]. ZIP8 was recently found as a novel molecular target in the treatment of neuroblastoma cancer. With the knockdown of the zinc transporter, realized by lentiviral-mediated RNA interference, there was a significant decrease in neuroblastoma cancer cells’ proliferation. In addition to this, the depletion of ZIP8 represses the capacity of migration of neuroblastoma cancer cells [30]. In astrocytes, the highest expression for zinc transporters was detected for *Slc30a9* (*ZnT9*) and *Slc39a1* (*Zip1*) (the two zinc transporters, compared with all others, were significantly higher expressed: *p* < 0.0001) (Figure 1B). In neurons, we have detected the prominent expression of *Slc30a1* (*ZnT1*), *Slc30a2* (*ZnT2*), *Slc30a3* (*ZnT3*), *Slc30a4* (*ZnT4*), *Slc30a5* (*ZnT5*), *Slc30a6* (*ZnT6*), *Slc30a7* (*ZnT7*), *Slc30a8* (*ZnT8*), *Slc30a10* (*ZnT10*), and *Slc39a1* (*Zip1*), *Slc39a2* (*Zip2*), and *Slc39a4* (*Zip4*) (we detected significant differences among means: *p* < 0.0001) (Figure 1C).

Based on these findings, we detected zinc transporters expressed uniquely in astrocytes, neurons, and zinc transporters expressed in neurons and astrocytes (Table 1).

Given that we found *Zip4* expressed in hippocampal brain lysate and hippocampal neuronal cultures and that the expression in neurons was significantly higher than that detected in astrocytes (Figure 1D), we next wanted to characterize further the so far unknown role of *Zip4* in nerve cells.

### 2.2. Zip4 Is Expressed and Localized at Glutamatergic Synapses in the Brain

**Figure 2:**
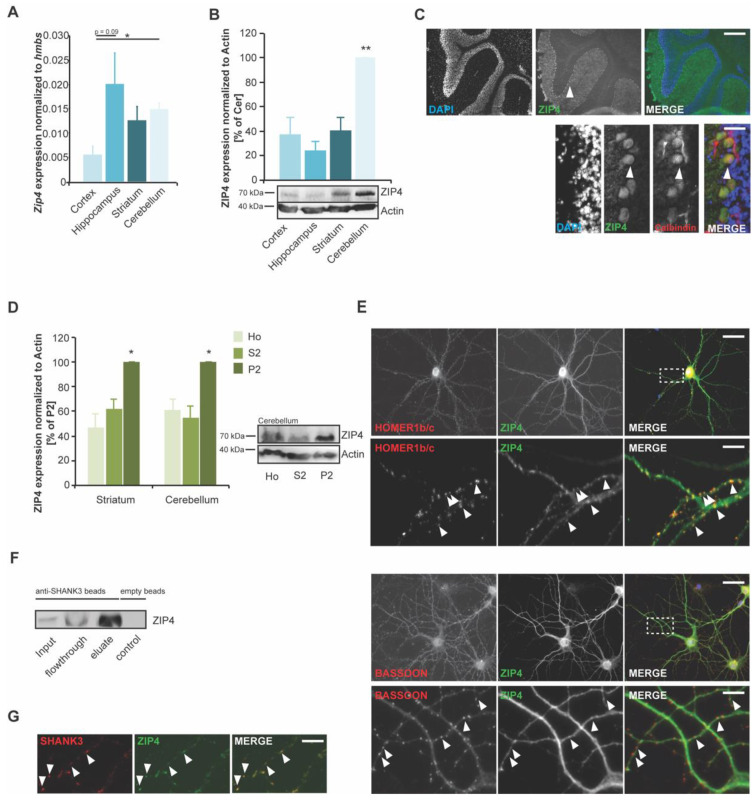
*Zip4* is expressed at synapses in neurons and enriched in Purkinje cells of the cerebellum. (**A**) Expression of the *Zip4* gene was detected in brain lysate of wild-type mice (*n* = 3 animals) and normalized against *hmbs*. Lowest mRNA levels were detected in cortex and significantly higher levels compared to cortex were found in cerebellum (ANOVA: F = 5.554; *p* = 0.006; posttest: ctx vs. cer *p* = 0.0144). (**B**) Expression of ZIP4 protein was similarly found in all analyzed brain regions (ctx, hip, str and cer), with highest levels observed in cerebellum (ANOVA: F = 15.440; *p* = 0.003; posttest: ctx vs. cer *p* = 0.017; hip vs. cer *p* = 0.006; str vs. cer *p* = 0.013) (*n* = 3 animals). (**C**) High ZIP4 expression was detected in Purkinje cells of the cerebellum. Images show representative stainings of mouse brain sections using ZIP4 antibody and calbindin as a marker for Purkinje neurons (arrows) (scale bar = 250 μm (upper panel) and = 30 μm (lower panel)). (**D**) ZIP4 expression is highest in the synapse associated protein fraction (P2), but ZIP4 is also found in the fraction containing soluble cytoplasmic proteins (cerebellum: ANOVA: F = 9.737, *p* = 0.0049, posttest: Ho vs. P2 *p* = 0.0354, S2 vs. P2 p = 0.0031; Striatum: Ho vs. P2 *p* = 0.0417, S2 vs. P2 *p* = 0.0468) (*n* = 3 per brain region and fraction). (**E**) Staining of primary hippocampal cultures at DIV14 shows that ZIP4 expression is found in neurons. MAP2 was used to visualize neuronal cells. DAPI was used to visualize cell nuclei. ICC confirms ZIP4 immunoreactive signals at synapses (arrows, zoomed region indicated by rectangle). There, ZIP4 signals colocalize with the postsynaptic marker protein HOMER1b/c (upper panel) but are juxtaposed to signals from the presynaptic marker protein BASSOON (lower panel) (scale bar = 50 μm (upper panels) and = 10 μm (lower panels)). (**F**) Co-IP experiments confirm that ZIP4 can be found in a complex centered around the postsynaptic scaffold protein SHANK3. SHANK3 was coupled to magnetic beads via SHANK3 antibody. Brain lysate from mice was used, and ZIP4 signal was detected in the input and in precipitated proteins in the eluate. Empty beads did not precipitate ZIP4. (**G**) Similar to colocalization with HOMER1b/c, ZIP4 signals colocalize with SHANK3 signals in hippocampal neurons in vitro (scale bar = 15 μm).

Given that *Zip4* has not been well characterized in the brain so far, we wanted to closer investigate its expression in the brain. To that end, we next used a qRT–PCR-based approach to quantify mRNA expression in technical triplicates using RNA lysate from four different brain regions (cortex (ctx), hippocampus (hip), striatum (str), and cerebellum (cer)) from 3 animals. In all brain regions, we detected *Zip4* mRNA. The lowest expression was observed in the cortex. Significantly higher levels compared to the cortex were found in the cerebellum and hippocampus (as a trend) (Figure 2A). Along with detecting mRNA, the expression of ZIP4 protein was found in all analyzed brain regions (Figure 2B). The highest levels were observed in the cerebellum. Given that mRNA and protein levels of ZIP4 were high in the cerebellum and that the detected expression may result from non-neural cells, such as cells of the blood capillary walls or blood cells in the tissue, we further assessed ZIP4 localization in the cerebellum. We detected the high and specific expression of ZIP4 in Purkinje neurons (Figure 2C) that were visualized by Calbindin staining, a marker for Purkinje neurons.

Next, we wanted to investigate the subcellular localization of ZIP4 in neurons. To that end, we performed protein fractionation of brain lysates to obtain, from homogenate, the S2 fraction containing soluble proteins and the P2 fraction (synaptosomal fraction), a fraction enriched in synaptic proteins. The results show that ZIP4 is enriched in the synaptosomal fraction (Figure 2D). Immunocytochemistry using primary neuronal cell cultures confirms the presence of ZIP4 in neurons. ZIP4 immunoreactive signals can be observed in the cell soma and also colocalizing with a synaptic marker protein (Homer1b/c) (Figure 2E upper panel). High-resolution images reveal a pattern, where the postsynaptic protein Homer1b/c is found closely colocalized with a ZIP4 signal, while ZIP4 is found in close juxtaposition to the presynaptic marker protein BASSOON (Figure 2E lower panel). These results hint towards a postsynaptic localization of ZIP4. To further confirm the postsynaptic expression, which is in line with the protein enrichment in the P2 fraction, we investigated whether it is possible to co-immunoprecipitate ZIP4 with a major scaffold protein of the postsynaptic density (PSD) of excitatory synapses, SHANK3. To that end, SHANK3 was coupled to magnetic beads and rat brain lysate incubated. While empty beads did not show ZIP4 signals in the eluate, SHANK3 coupled beads were able to precipitate ZIP4 (Figure 2F). Similar to the results obtained for HOMER1b/c, a direct interaction partner of SHANK3, ZIP4 signals also colocalize with SHANK3 signals (Figure 2G). Taken together, the data shows that ZIP4 is a zinc transporter that is present at postsynapses of neuronal cells with prominent expression in Purkinje cells of the cerebellum but also found in a postsynaptic complex with SHANK3 in excitatory hippocampal neurons.

### 2.3. ZIP4 Expression Is Sensitive to Local Zinc Levels

**Figure 3:**
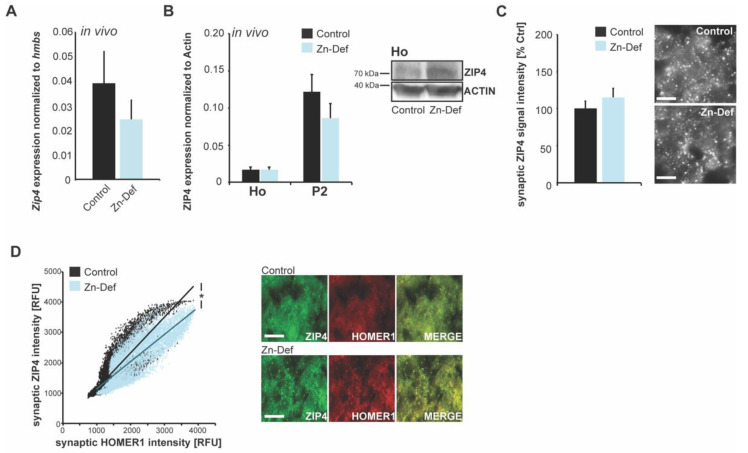
Only synaptic ZIP4 expression is sensitive to local zinc levels. (**A**) Expression of the *Zip4* gene was detected in whole brain lysate of wild-type mice (*n* = 3 mice) and acute zinc-deficient mice (*n* = 3 mice) and normalized against *hmbs*. No significant difference was detected between control and zinc-deficient mice (*t*-test). (**B**) Acute zinc-deficient mice also showed no significant change of ZIP4 on protein level in whole-brain homogenate and P2 fractions compared to controls (*n* = 3 per group). (**C**) No significant change of ZIP4 on protein level was detected using immunohistochemistry. The average signal intensity of ZIP4-positive puncta was measured in the cerebellum of control and acute zinc-deficient mice (t-test, *n* = 3 mice per group) (scale bar = 5 μm). (**D**) A co-staining of HOMER1b/c was performed and the ZIP4 signal intensity at HOMER1b/c-positive puncta correlated with HOMER1b/c signal intensity in the cerebellum of mice. Acute zinc-deficient mice show a significant reduction of ZIP4 signal intensity that positively correlates with HOMER1b/c signal intensity (R-values were obtained with the Spearman correlation r_Ctrl_ = 0.774; *p*_Ctrl_ < 0.0001; *r*_Zn-def_ = 0.844; *p*_Zn-def_ < 0.0001). Fisher-Z transformation reveals a significant loss at puncta with higher HOMER1b/c signal intensities (z_Ctrl_ = 1.030; z_Ctrl_ = 1.235; σ = 0.014; Z = 14.402 with no overlap of the 95%—confidence intervals, *n* = 3 mice per group) (scale bar = 10 μm).

In the intestinal system, where ZIP4 serves as a major zinc importer, both the level of *Zip4* mRNA abundance and subcellular localization of ZIP4 change in response to Zn levels [11]. Therefore, in the next set of experiments, we evaluated whether neuronal *Zip4* is also sensitive to changes in systemic zinc concentrations. To that end, we used tissue from acute zinc-deficient mice with systemic zinc deficiency and lower brain zinc levels [31,32]. We measured the expression of the *Zip4* gene in the whole-brain lysate of control and acute zinc-deficient mice (Figure 3A). We could not detect a significant difference between control and zinc-deficient mice. Further, no alteration in ZIP4 concentration was detected both in whole-brain homogenate and synapse enriched (P2) fractions on protein level in zinc-deficient mice (Figure 3B).

As ZIP4 expression was previously found pronounced in the cerebellum, we measured the intensity of ZIP4 immunoreactive signals correlating with protein levels by immunohistochemistry. Again, we could not detect a significant difference between control and acute zinc-deficient mice (Figure 3C). ZIP4 signals were analyzed as HOMER1b/c colocalizing, putative excitatory synaptic puncta. A correlation of ZIP4 and HOMER1b/c signal intensity at corresponding sites reveals that although the overall signal intensity of ZIP4 is not significantly different in zinc-deficient mice compared to controls, a specific and significant loss occurs at synapses with higher HOMER1b/c signal intensity, probably labeling the pool of mature synapses (Figure 3D).

### 2.4. Synaptic Expression of ZIP4 Shows Dynamic Responses to Transient Local Changes in Zinc Concentrations

**Figure 4:**
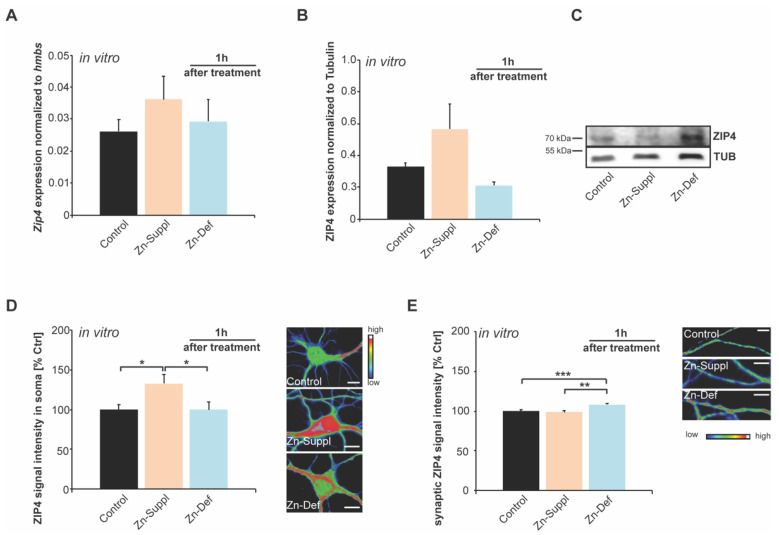
Primary hippocampal neurons analyzed 1 h after treatment with 30 µM ZnCl_2_ (Zn suppl.) or 30 µM TPEN (Zn-def.) and compared to untreated controls. (**A**) Expression of the *Zip4* gene was detected in cell lysate. No significant differences were detected after 1 h (one-way ANOVA) (*n* = 3 cultures per group). (**B**,**C**) Using protein lysate from these cells, expression of the ZIP4 protein was detected 1 h after treatments and compared to untreated controls. No significant change was detected (one-way ANOVA: *p* = 0.092) (*n* = 3 per condition). (**D**,**E**) The intensity of ZIP4 immunoreactive signals was measured in 10 cells per condition. (**D**) The intensity of immunoreactive signals in the cell soma is significantly increased 1 h and zinc supplementation (one-way ANOVA; *p* = 0.005; Tukey posttest: control vs. Zn_Sup_ *p* = 0.0222; Zn_Sup_ vs. Zn_Def_ *p* = 0.0376) (scale bar = 10 μm). (**E**) The signal intensity of immunoreactive puncta along dendrites is significantly increased 1 h after induction of zinc deficiency (one-way ANOVA: F_2,25_ = 7.378; *p* = 0.003; Tukey posttest: control vs. Zn_Def_ *p* = 0.0006; Zn_Suppl_ vs. Zn_Def_ *p* = 0.0059) (scale bar = 10 μm).

To simulate a more local, temporary, and short-term change in zinc levels, we further used primary hippocampal neurons treated one hour with TPEN to deplete zinc levels and with ZnCl_2_ to increase the zinc concentration in the medium. Similar to the results from zinc-deficient mice, we could not detect altered mRNA expression of the *Zip4* gene in response to lowered zinc levels one hour after the treatment (Figure 4A). To investigate whether changes occur on the protein level, we further analyzed protein lysate obtained from treated hippocampal neurons (whole-cell homogenate). No significant differences were detected on the whole-cell level after one hour of treatment (Figure 4B,C). To understand whether specific subcellular pools of ZIP4 are affected by the change in local zinc concentration, we performed immunocytochemistry experiments. We could detect ZIP4-positive signals along the dendrites that most likely represent synaptic signals, as well as ZIP4-positive signals in the soma of neurons. The intensity of immunoreactive signals in the cell soma is significantly increased one hour after zinc supplementation (Figure 4D), while the signal intensity of immunoreactive puncta along dendrites is not altered (Figure 4E). However, under zinc-depleted conditions, the dendritic signal intensity of ZIP4 increases (Figure 4E). This somatic ZIP4 signal is increased in response to increasing zinc levels. Still, dendritic ZIP4 levels are increased in response to decreasing zinc levels hinting at two distinct roles of ZIP4 in neurons based on the transporter’s localization.

## 3. Discussion

The importance and complexity of zinc signaling in the body are highlighted by the existence of 24 zinc transporters that, together with several zinc-buffering proteins, maintain specific zinc homeostasis. Within the rat brain tissue, we detected the expression of all zinc transporters. However, brain tissue is comprised of many different cells, including, for example, endothelial cells and blood cells. Therefore, we have further investigated zinc transporter expression utilizing rat neuronal and astrocyte cell cultures. We have found major differences in expression levels and distribution of zinc transporters between neurons and glial cells, such as astrocytes hinting at particular roles of some of the zinc transporters within the brain.

We found neuronal expression of *ZnT1–8*, *ZnT10*, as well as *Zip1*, *Zip2* and *Zip4*. A role for ZnT1 has been proposed as a synaptic interaction partner of NMDA receptors [3], and ZnT3 is well characterized as a presynaptic vesicular protein importing zinc into neurotransmitter vesicles [2]. *ZnT2* expression was previously reported in neuronal stem cells [33], and ZnT2 may be localized to neuronal mitochondria, but a particular role in neurons has not been reported so far. Abundant *ZnT4* expression was detected in the brain, and *ZnT5* is known to be ubiquitously expressed, but similar to *ZnT7* and *ZnT8*, their role in neurons has not been well investigated so far. *Zip1* mRNA expression was found in several brain regions, including the hippocampus, cerebellum, and thalamus and associated with neuronal cell bodies [5], where it may function as major endogenous zinc uptake transporter. However, isoforms of ZIP1 and ZIP2 also bind to both K_v_beta2 subunits of potassium channels and protein kinase C (PKC) zeta and are regulated by neurotrophic factors [34]. Intriguingly, we confirmed the presence of *Zip4* detected in brain tissue, in cell cultures, and at significantly higher expression levels in neurons.

Zinc is one of the most abundant trace metals in the brain [1]. On a cellular level, in neurons, zinc is diffusely distributed in the cytoplasm and nucleus [35]. However, zinc is also found in dendrites and axons of neurons and is enriched at synaptic terminals [36,37]. While zinc is especially enriched in the hippocampus and cerebral cortex in the adult brain, during neonatal development, the highest zinc levels can be found in the cerebellum. This is accompanied by the rapid growth of the cerebellum during the development of motor skills. Here, we have detected ZIP4 on mRNA and protein levels in all analyzed brain regions, including the hippocampus and cerebellum, with the highest levels in the cerebellum. In particular, we detected strong expression in Purkinje cells. In the adult brain, only low levels of chelatable (synaptic) zinc are found in the cerebellum, and there is little evidence that zinc is released from cerebellar neurons. However, in acutely isolated Purkinje cells, GABA currents were sensitive to zinc [38]. Thus, it has been speculated that besides ZnT3, the principal regulator of vesicular zinc, whose expression is low in the cerebellum, different types of zinc transporters found in the cerebellum may play a physiological role [39]. Interestingly, the addition of nitric oxide donors causes large increases in the intracellular concentration of zinc in Purkinje cells [40].

Within neurons, we detected a somatic pool of ZIP4 and ZIP4 present along the cells’ dendrites. These dendritic signals colocalized with two scaffold proteins of the hippocampal excitatory PSD, namely HOMER1 and SHANK3. Immunoprecipitation experiments showed that ZIP4 is part of the same protein complex. An interaction between ZIP4 and SHANK3 has been reported before, however, outside of the brain in the gastrointestinal system. Within enterocytes, similarly, ZIP4 and SHANK3 were co-immunoprecipitated [41]. The localization of ZIP4 at synapses likely allows for a similar protein complex to form, thereby targeting ZIP4 to PSDs.

Future research needs to determine the exact role of ZIP4 at synapses and its physiological relevance. We did not detect significant responses of neuronal *Zip4* expression levels to systemic chronic zinc deficiency. Therefore, in the brain, ZIP4 may have a more specialized function than regulating cellular zinc levels in general. In line with this hypothesis, we found that chronic zinc deficiency affects only a specific pool of synaptic ZIP4 proteins found at synapses with high HOMER1 levels, likely the pool of mature synapses [42]. In contrast, acute short-term changes in zinc levels lead to an increase in synaptic ZIP4 protein levels under low-zinc conditions, while somatic ZIP4 levels are higher in response to increased zinc availability. Recently, it has been shown that ZIP4 acts as a zinc sensor. Zinc binding at concentrations of around 10 μM triggers conformation-dependent endocytosis of the zinc transporter [43]. However, local extracellular zinc concentrations within the synaptic cleft may reach more than 1 mM [44]. In our experiment, we did not observe a significant decrease of synaptic ZIP4 after zinc supplementation. This may be due to ZIP4 proteins remaining synaptic and being endocytosed at the synaptic spine. In contrast, zinc deficiency resulted in an increased ZIP4 signal at synapses, arguing for a synaptic ZIP4 regulation by yet-to-be-identified zinc-dependent proteins.

Given that ZIP4 is a zinc importer, it seems possible that ZIP4 is involved in local zinc import at synapses. It has been shown that zinc is co-released with glutamate in the hippocampus, especially at mossy fiber terminals in the CA3 region. Subsequently, it can enter the postsynaptic compartment through AMPA and NMDA receptors and Ca_V_ channels [45,46,47]. ZIP4 may allow further modulation of this process. In the cerebellum, where little synaptic zinc occurs, ZIP4 may even be a vital contributor to regulating the local synaptic zinc levels. Several proteins in the postsynaptic compartment are zinc-binding proteins, among them SHANK2 and SHANK3 [48], and zinc is vital for the assembly of PSDs [49]. Moreover, local synaptic zinc levels modulate postsynaptic plasticity with zinc concentrations directly correlating with PSD scaffold strength [48,50,51]. Therefore, there is a need for carefully controlling local zinc import and export.

Evidence is mounting that trace metals, such as iron, zinc, and copper, are involved in pathomechanisms of Alzheimer’s disease (AD) [52]. Zinc binds to amyloid-beta peptides and thereby is sequestered into senile plaques, resulting in low synaptic zinc availability and, thereby, loss of excitatory synapses [53]. Thus, identifying a novel synaptic zinc transporter may reveal a so-far underexplored drug target for the treatment of AD, where influencing zinc concentrations showed promising effects in the past [54].

## 4. Materials and Methods

### 4.1. Materials

Primary antibodies were purchased from Abcam (Cambridge, UK) (calbindin, cat. no. ab82812: IHC: 1:200; MAP2, cat. no. EPR19691: ICC: 1:500), Covance (Princeton, NJ, USA) (βIII-tubulin, cat. no. PRB-435P: WB 1:5000), OriGene (Herford, Germany) (ZIP4, cat. no. TA333766: ICC 1:500, WB 1:500), Sigma-Aldrich (Arklow, Ireland) (β-actin, cat. no. A2228: WB 1:10000), Synaptic systems (BASSOON, cat. no. 141004: ICC 1:500; HOMER1b/c, cat. no. 160022: ICC 1:500, IHC: 1:200) and Thermo Fisher Scientific (ZIP4, cat. no. PA5-101971: ICC 1:150, IHC 1:200, WB 1:1000). SHANK3 antibodies (ICC 1:500, WB 1:500) have been described previously [32]. Secondary Alexa-coupled antibodies were purchased from Invitrogen and HRP-coupled from Dianova and Dako. Secondary-HRP conjugated antibodies were purchased from Thermo Fisher (Thermo Fisher Scientific, Waltham, MA, USA). Unless otherwise indicated, all other chemicals were obtained from Sigma-Aldrich (Merck, Ireland).

### 4.2. Animals

Pregnant rats (Sprague Dawley) were used for brain tissue analyses. In addition, tissue was used from acute zinc deficient and control mice (female C57BL/6JRj mice) that were generated previously [31]. In brief, animals were housed upon arrival in the animal facility in plastic cages under standard laboratory conditions and provided with food and water available ad libitum. Mice (4 weeks of age) were habituated for one week (receiving control diet (35 ppm zinc)). Subsequently, one group of mice received a special laboratory diet (ssniff GmbH, Soest, Germany (4 ppm zinc)) with access to food and water ad libitum for 9 weeks. All animal experiments were performed following the guidelines and regulations for the welfare of experimental animals issued by the Federal Government of Germany and by the local ethics committee (Ulm University) ID nos. O.103 and 1257. The protocol used was approved by the Regierungspräsidium Tübingen, the state of Baden-Württemberg, and the ethics committee of Ulm University.

### 4.3. Preparation and Cultivation of Primary Hippocampal Neurons

Primary hippocampal neuron cultures were prepared, starting from rat brain tissue (sacrificed in the embryonic day 18; E18) obtained from BrainBits (BrainBits LLC, Springfield, IL, USA) following the protocol described previously [55].

In detail, the tissue samples were washed with Hanks’ balanced salts solution (HBSS, Carl Roth GmbH + Co. KG, Karlsruhe, Germany). After that, a cell suspension was obtained by treating the tissue with 0.25% trypsin (Sigma-Aldrich, Arklow, Ireland) for 20 min at 37 °C, followed by homogenization. After this step, the sample was diluted with Dulbecco’s modified Eagle’s medium, 10% fetal bovine serum, 2 mM L-glutamine, 100 U/mL penicillin, and 100 μg/mL streptomycin. The neurons were then plated on 24 well plates with glass coverslips coated with 0.1 mg/mL poly-L-lysine at a density of 3 × 10^4^ cells or 10 cm Petri dishes with a density of 3 × 10^6^. The neurons were incubated overnight (37 °C, 5% CO_2_), and after 24 h, the plating medium (DMEM) was exchanged for neurobasal medium, including 0.5 mM L-glutamine, 2% B27 supplement, and antibiotics. The primary hippocampal neurons were cultured at 37 °C and 5% CO_2_ for 14 days.

### 4.4. Cultivation of Cell Lines

DI TNC1 (ATCC, Manassas, VA, USA: CRL-2005) rat astrocytes were seeded (50,000 cells/mL) on poly-L-lysine (0.1 mg/mL)-coated 10 cm Petri dish and 24 multiwell and incubated in Dulbecco’s modified Eagle’s medium (DMEM) containing 10% fetal bovine serum, 2% glutamine, 1% sodium pyruvate and 1% penicillin–streptomycin for 3 days until they were confluent. The culture was incubated at 37 °C in a suitable incubator with 5% CO_2_.

C6 glioblastoma (ATCC, Manassas, VA, USA: CCL-107) rat glioblastoma cells were seeded (50,000 cells/mL) on poly-L-lysine (0.1 mg/mL)-coated 10 cm Petri dish and 24 multiwell and incubated in F-12K nutrient mixture (1X) containing 20% fetal bovine serum, 1% penicillin–streptomycin for 72 h until they reach the 80–85% confluence. The cells were incubated at 37 °C in a suitable incubator with 5% CO_2_ in an air atmosphere.

### 4.5. Immunocytochemistry (ICC)

For ICC, the cells were washed with 1X PBS and fixed with 4% PFA containing 4% sucrose for 20 min at 4 °C. After removing the PFA, the cells were washed with 1X PBS three times; each washing step lasted for 5 min at RT. The cells were incubated with blocking solution (BS) (10% FBS/1× PBS) for 1 h at RT to avoid unspecific binding of antibodies. Afterward, cells were incubated with the primary antibody in BS overnight at 4 °C, followed by three washing steps with 1X PBS to wash out the unbound primary antibody. The secondary antibody was diluted in BS and applied for 1 h at RT. Afterward, the cells were washed with water to avoid salt crystals. Finally, the cells were mounted with ProLong^TM^ gold antifade mountant (Thermo Fisher/BioSciences, Dublin, Ireland) with DAPI to counterstain the cell nuclei.

### 4.6. Immunohistochemistry (IHC)

Cryosections (thickness 14 µM) were thawed and fixed in paraformaldehyde (PFA) for 20 min in a hydrated box. Then the sections were washed with 1× PBS for 10 min, followed by permeabilization with 0.2% triton in 1× PBS for 2 h. Afterward, the slices were washed again with 0.05% triton in 1× PBS for 10 min. Later blocking of antigens was done using incubation with 10% FCS for 1.5 h. Then, the slices were incubated with primary antibody diluted in blocking solution at 4 °C overnight. Subsequently, the slices were washed three times with 0.05% triton in 1× PBS for 10 min each. The slices were incubated with a secondary antibody diluted in blocking solution at 37 °C for 1.5 h in the dark. After washing with 1× PBS containing 0.05% triton, the slices were washed two times with PBS and incubated 5 min with PBS containing DAPI, and after a final washing with ddH_2_O for 5 min, slices were mounted with VectaMount (Vector Laboratories).

### 4.7. Cell Lysis and Total RNA Preparation

To purify RNA from brain tissue, brains were removed and snap-frozen in liquid nitrogen. The tissue was thawed for 10 min and homogenized in 1 mL Qiazol lysis reagent (Qiagen, Manchester, UK). QIAshredder columns were loaded and centrifuged at 12,000× *g* for 2 min at 4 °C. Afterward, 200 μL of chloroform was added, and the mixture was shaken vigorously for 15 s. After incubation at RT for 2–3 min, the samples of hippocampi were centrifuged at 12,000× *g* for 15 min at 4 °C. The upper phase, the aqueous phase containing the RNA, was transferred to a new reaction tube and mixed in ratio 1:1 volume (usually 600 μL) of 70% Ethanol (EtOH). Successively, RNeasy spin columns were used for the following steps, and the RNA was purified using the RNeasy lipid tissue mini kit (Qiagen, Manchester, UK) according to the manufacturer’s protocol.

C6 glioblastoma and DI TNC1 astrocyte cells were grown on a Ø 10 cm Petri dish at a density of 5 × 10^4^ cells/mL and primary cultures of hippocampal neurons DIV14 grown on a Ø 10 cm Petri dish at a density of 3 × 10^6^, respectively; cells were washed with ice-cold 1X PBS. After adding buffer RLT (350 μL), the cells were transferred into a 1.5 mL tube using a plastic cell scraper. The following steps were performed using the RNeasy mini kit (Qiagen, Manchester, UK) according to the manufacturer’s protocol. All additional washing steps were carried out, and RNA was eluted in a final volume of 30 μL. The purity of the samples was analyzed at the BioTek Nanodrop (BioTek Instruments, Winooski, VT, USA).

### 4.8. qRT–PCR

For qRT–PCR experiments, three biological replicates (animals, cell cultures) were used, the RNA extracted separately, and the samples were run in technical triplicates each. For quantitative measurement of mRNA levels, first-strand synthesis and quantitative real time-PCR amplification was carried out in a one-step, using the QuantiFast^®^ primer assay for SYBR green-based real time RT–PCR (Qiagen, Manchester, UK). Amplification of cDNA was monitored and analyzed by measuring the intercalating of the fluorescence dye SYBR Green I in double-stranded DNA. Primers were commercially available and validated primer sets (Qiagen, QuantiTect primer assays: Rn_Slc30a1_2_SG, cat. no. QT01084461; Rn_Slc30a2_1_SG, cat. no. QT00181237; Rn_Slc30a3_1_SG, cat. no. QT00380443; Rn_RGD: 619750_1_SG, cat. no. QT00192465; Rn_Slc30a5_1_SG, cat. no. QT01589322; Rn_Slc30a6_1_SG, cat. no. QT01573509; Rn_Slc30a7_1_SG, cat. no. QT01572179; Rn_Slc30a8_1_SG, cat. no. QT00471912; Rn_Slc30a9_1_SG, cat. no. QT01590050; Rn_Slc30a10_1_SG, cat. no. QT01594985; Rn_Slc39a1_1_SG, cat. no. QT01585080; Rn_Slc39a2_1_SG, cat. no. QT01294153; Rn_Slc39a3_1_SG, cat. no. QT01584681; Rn_Slc30a4_1_SG, cat. no. QT01624889; Rn_Slc39a5_1_SG, cat. no. QT01610084; Rn_Slc39a6_1_SG, cat. no. QT01583785; Rn_Slc39a7_1_SG, cat. no. QT00437059; Rn_Slc39a8_1_SG, cat. no. QT01599584; Rn_Slc39a_1_SG, cat. no. QT01598786; Rn_Slc39a10_1_SG, cat. no. QT01619940; Rn_Slc39a11_1_SG, cat. no. QT01575896; Rn_Slc39a12_1_SG, cat. no. QT01624364; Rn_Slc39a13_1_SG, cat. no. QT01575385; Rn_Slc39a_1_SG, cat. no. QT01084461; Rn_Slc39a14_1_SG, cat. no. QT01618155; Rn_HMBS_1_SG, cat. no. 249900).

In this type of quantitative PCR, reverse transcription and amplification are realized in the same reaction. Total RNA was reversely transcribed and subsequently amplified using the 2× QuantiFast SYBR^®^ green RT–PCR master mix (Qiagen, Manchester, UK). About 1 μM of both sense and antisense primers were added. Internal standards (housekeeping gene) and samples were amplified simultaneously according to the manufacturer’s protocol.

Thermal cycling and fluorescent detection were performed using the QuantStudio™ 7 Flex Real-Time (model 4485695) (Thermo Fisher Scientific, Waltham, MA, USA). Relative quantification is based on internal reference genes to determine virtual mRNA levels of the target gene. Virtual mRNA levels of different target genes were shown normalized against virtual mRNA levels of the internal housekeeping gene hydroxymethylbilane synthase (*hmbs*). Cycle threshold (ct) values were calculated by the QuantStudio™ 7 Flex Real-Time (version 1.3). All quantitative real-time PCR reactions were run in technical triplicates, and mean ct-values for each reaction were taken into account for the calculations. The threshold fluorescence to detect cDNA was set at 0.01. Further, the given ct values were transformed into virtual mRNA levels according to the following formula:

virtual mRNA level = 10 ct (target) − ct (standard)/slope of standard curve(1)

The ct of the standard curve was defined as 15, and the slope of the standard curve was defined as −3.33. The target gene’s virtual mRNA level was then normalized against the virtual mRNA level of the housekeeping gene (*hmbs*).

### 4.9. Protein Biochemistry

Western blot experiments were performed using homogenate, S2 fractions, and PSD-enriched P2 fractions. Brains/brain regions were isolated from animals and were dissolved in HEPES buffer (10 mL buffer/gram tissues) (10 mM HEPES; 0.32 M sucrose, pH 7.42; protease inhibitor cocktail tablet (Roche)). Tissues were homogenized using sonication to obtain crude homogenate. The crude homogenates were centrifuged at 3200 rpm for 15 min at 4 °C, resulting in the nuclear fraction (P1) and soluble supernatant (S1). The S1 fractions were further centrifuged at 11,400 rpm for 20 min at 4 °C. Subsequently, the pellet or synaptosomal fraction (P2) was dissolved in ice-cold HEPES buffer. The protein concentration was measured using Bradford analysis, and 10, 30, or 45 µg of protein was loaded on PAGE in 4x SDS sample-loading buffer. Proteins were separated by SDS–PAGE and blotted onto nitrocellulose membranes. Immunoreactivity was visualized using HRP- conjugated secondary antibodies and the SuperSignal detection system (Pierce, Upland, IN, USA).

PSD-fractionation: postsynaptic density protein (PSD) from female adult hippocampus brains were isolated in the following way. The hippocampus was homogenized in ice-cold buffer-1 (10 mM HEPES, pH 7.4, 2 mM EDTA, 5 mM sodium orthovandate, 30 mM sodium fluoride, 20 mM ß-glycerol phosphate) using a pulse sonicator (Fisher Scientific, Waltham, MA, USA). Cell debris and nuclei were removed by centrifugation at 500× *g* for 5 min at 4 °C, obtaining supernatant S1 (homogenate: cytosolic and membrane-associated fraction) and removing pellet P1 (nuclei-enriched fraction). In a second moment, the supernatant S1 was centrifuged at 10,000× *g* at 4 °C resulting in supernatant S2 (cytosolic compartment fraction) and sample P2 (crude-membrane fraction). The sample P2 was resuspended in buffer-2 (50 mM HEPES, pH 7.4, 2 mM EDTA, 2 mM EGTA, 5 mM sodium orthovandate, 30 mM sodium fluoride, 20 mM ß-glycerol phosphate, 1% Triton X). The fraction P2 was centrifuged at 20,000 × *g* for 80 min at 4 °C resulting in supernatant S3 (synaptic cytosol) and fraction P3 (synaptosomes and PSD fraction). As the last step, the fraction P3 was resuspended in buffer-3 (50 mM Tris, pH 9, 5 mM sodium orthovandate, 30 mM sodium fluoride, 20 mM ß-glycerol phosphate, 1% NaDOC). The protein concentration of the brain tissue homogenates was determined using the Bradford assay.

Western blotting (WB): Proteins were separated by SDS–PAGE and blotted onto nitrocellulose membranes. Immunoreactivity was visualized using HRP-conjugated secondary antibodies using the Pierce™ ECL Western blotting substrate (Thermo Fisher Scientific, Waltham, MA, USA).

WB quantification: Evaluation of bands from WBs was performed using ImageJ. The experimental blots were imaged using an Alliance Q9 Advanced from Uvitec.

Co-immunoprecipitation: For co-immunoprecipitation (Co-IP) experiments, whole-cell lysates were used for immunoprecipitation using the μMACS magnetic bead separation system (Miltenyi Biotec). The magnetic beads were loaded with Shank3 antibodies.

### 4.10. Statistics

Signal intensities—Fluorescence images were obtained using an upright Axioscope microscope equipped with a Zeiss CCD camera (16 bits; 1280 × 1024 pixels per image) using the AxioVision software (Zeiss) with the same exposure time throughout the experiment and all of the conditions and were analyzed using ImageJ 1.51 a. Statistical analysis was performed using Microsoft Excel for Windows and tested for significance using unpaired *t*-tests or ANOVA. All values were normally distributed.

Synapse density—Primary hippocampal neurons were stained with Homer1b/c as a postsynaptic protein or Bassoon as a presynaptic marker. Images of 10 neurons per condition were obtained in a 40× magnification with the same exposure time throughout the experiment. The number of synaptic proteins was measured along primary dendrites. For each measurement, the number of synapses per 100 pixels was calculated. Statistical analysis was performed using Microsoft Excel for Windows and tested for significance using unpaired *t*-tests or ANOVA. All values were normally distributed.

All other statistical analysis was performed with GraphPad Prism 8 Version 8.4.1 (460). Data are shown as mean ± standard error of the mean (SEM). For comparisons, analysis of variance (ANOVA) was performed, followed by post hoc tests for within-group comparisons (Tukey’s or Bonferroni tests). For comparisons of two independent groups, Student’s *t*-tests were used. Statistically significant differences are indicated in figures by * *p* ≤ 0.05, ** *p* ≤ 0.01, and *** *p* ≤ 0.001.

## Figures and Tables

**Table 1:** List of zinc transporters that are expressed in a cell type-specific manner.

Cell Type	ZnT	Zip
Neurons	*ZnT1, ZnT2, ZnT3, ZnT4, ZnT5, ZnT6, ZnT7, ZnT8, ZnT10*	*Zip1, Zip2, Zip4*
Astrocytes	*ZnT1, ZnT4, ZnT5, ZnT6, ZnT7, ZnT9*	*Zip1, Zip6, Zip8 ^1^, Zip9, Zip10, Zip11, Zip13*

^1^ significantly higher in glioblastoma cells (unpaired *t*-test: *p* = 0.038).

## Data Availability

The data presented in this study are available in de Benedictis, C.A.; Haffke, C.; Hagmeyer, S.; Sauer, A.K.; Grabrucker, A.M. Expression Analysis of Zinc Transporters in Nervous Tissue Cells Reveals Neuronal and Synaptic Localization of ZIP4. *Int. J. Mol. Sci.* **2021**, *22*, x. https://doi.org/10.3390/ijms22094511.

## Supplementary Materials

Supplementary Materials can be found at https://www.mdpi.com/article/10.3390/ijms22094511/s1.

## Abbreviations

cer	Cerebellum
ctx	Cortex
DIV	Days in vitro
hip	Hippocampus
HRP	Horseradish peroxidase
RT	Room temperature
str	Striatum
TPEN	N,N,N′,N′-tetrakis(2-pyridinylmethyl)-1,2-ethanediamine
