# Supplementary material for: Expression Analysis of Zinc Transporters in Nervous Tissue Cells Reveals Neuronal and Synaptic Localization of ZIP4
Source: Int J Mol Sci. 2021 Apr 26;22(9):4511. doi: 10.3390/ijms22094511 (PMC8123391; doi:10.3390/ijms22094511)
Supplement: Supplementary file 1 [file ijms-22-04511-s001.zip › ijms-1153872-supplementary.pdf]

International Journal of
Molecular Sciences

MDPI

Supplementary Information

Expression Analysis of Zinc Transporters in Nervous Tissue Cells Reveals Neuronal and Synaptic Localization of ZIP4

Chiara A. De Benedictis, Claudia Haffke, Simone Hagmeyer, Ann Katrin Sauer, Andreas M. Grabrucker

Supplementary table S1: **A)** In rat lysate from rat hippocampi, we found the expression of ZnT transporters relevant from the statistical analysis (one-way ANOVA: p=0.0049). Post-hoc tests (Tukey's multiple comparisons test), underlined the following results: *ZnT1* vs. *ZnT2* (p=0.0218), *ZnT2* vs. *ZnT4* (p=0.0188), *ZnT2* vs. *ZnT7* (p=0.0468), *ZnT2* vs. *ZnT9* (p=0.0061). The Zip transporters expressed in the lysate of hippocampal lysates were not statistically significant (one-way ANOVA: p=0.0576). **B)** In C6 Glioblastoma cell line, on mRNA level, we detected the expression of the following ZnTs, with significant differences among means after ANOVA and post-hoc tests: *ZnT1* vs. *ZnT9* (p<0.0001), *ZnT4* vs. *ZnT9* (p<0.0001), *ZnT5* vs. *ZnT9* (p<0.0001), *ZnT6* vs. *ZnT9* (p<0.0001), *ZnT7* vs. *ZnT9* (p<0.0001). The Zips significantly expressed in C6 Glioblastoma were: *Zip1* vs. *Zip9* (p<0.0001), *Zip1* vs. *Zip10* (p=0.0183), *Zip1* vs. *Zip13* (p<0.0001), *Zip6* vs. *Zip9* (p<0.0001), *Zip6* vs. *Zip13* (p=0.0305), *Zip9* vs. *Zip10* (p=0.0006). In the DI TNC1 Astrocytes cell line we found the expression of the following ZnTs statistically significant different (ANOVA: p<0.0001, Tukey's multiple comparisons test): *ZnT1* vs. *ZnT7* (p<0.0001), *ZnT1* vs. *ZnT9* (p<0.0001), *ZnT4* vs. *ZnT6* (p=0.0047), *ZnT4* vs. *ZnT7* (p=0.0133), *ZnT4* vs. *ZnT9* (p<0.0001), *ZnT5* vs. *ZnT7* (p=0.0001), *ZnT5* vs. *ZnT9* (p<0.0001), *ZnT6* vs. *ZnT7* (p<0.0001), *ZnT6* vs. *ZnT9* (p<0.0001), *ZnT7* vs. *ZnT9* (p<0.0001). The following levels of expression of several Zip transporters were significantly different (Tukey's multiple comparisons test): *Zip1* vs. *Zip8* (p<0.0001), *Zip1* vs. *Zip9* (p<0.0001), *Zip1* vs. *Zip10* (p<0.0001), *Zip1* vs. *Zip11* (p<0.0001), *Zip1* vs. *Zip13* (p<0.0001), *Zip6* vs. *Zip8* (p<0.0001), *Zip6* vs. *Zip9* (p<0.0001), *Zip6* vs. *Zip10* (p=0.0048), *Zip6* vs. *Zip11* (p<0.0001), *Zip6* vs. *Zip13* (p=0.0019), *Zip8* vs. *Zip11* (p=0.0134), *Zip10* vs. *Zip11* (p<0.0001), *Zip11* vs. *Zip13* (p<0.0001). **C)** In primary hippocampal neurons, ANOVA and post-hoc Tukey's multiple comparisons reveled significant differences: *ZnT1* VS. *ZnT7* (p<0.0001), *ZnT1* vs. *ZnT10* (p=0.0083), *ZnT2* vs. *ZnT7* (p<0.0001), *ZnT3* vs. *ZnT7* p<0.0001), *ZnT3* vs. *ZnT10* (p=0.0051), *ZnT4* vs. *ZnT7* (p<0.0001), *ZnT4* vs. *ZnT10* (p=0.0051), *ZnT5* vs. *ZnT7* (p<0.0001), *ZnT5* vs. *ZnT10* (p<0.0055), *ZnT6* vs. *ZnT7* (p<0.0001), *ZnT7* vs. *ZnT8* (p<0.0001), *ZnT7* vs. *ZnT10* (p=0.0005), *ZnT8* vs. *ZnT10* (p=0.0050). Regarding the Zip solute carrier family, we detected the following significant differences: *Zip1* vs. *Zip2* (p=0.0076), *Zip1* vs. *Zip4* (p=0.0035), *Zip2* vs. *Zip4* (p<0.0001).
